# Supplementary material for: Tumor-Informed Approach Improved ctDNA Detection Rate in Resected Pancreatic Cancer
Source: Int J Mol Sci. 2022 Sep 29;23(19):11521. doi: 10.3390/ijms231911521 (PMC9570468; doi:10.3390/ijms231911521)
Supplement: Supplementary file 1 [file ijms-23-11521-s001.zip › Panc_feasibility_Supplement figure_revise.pptx]

## Slide 1
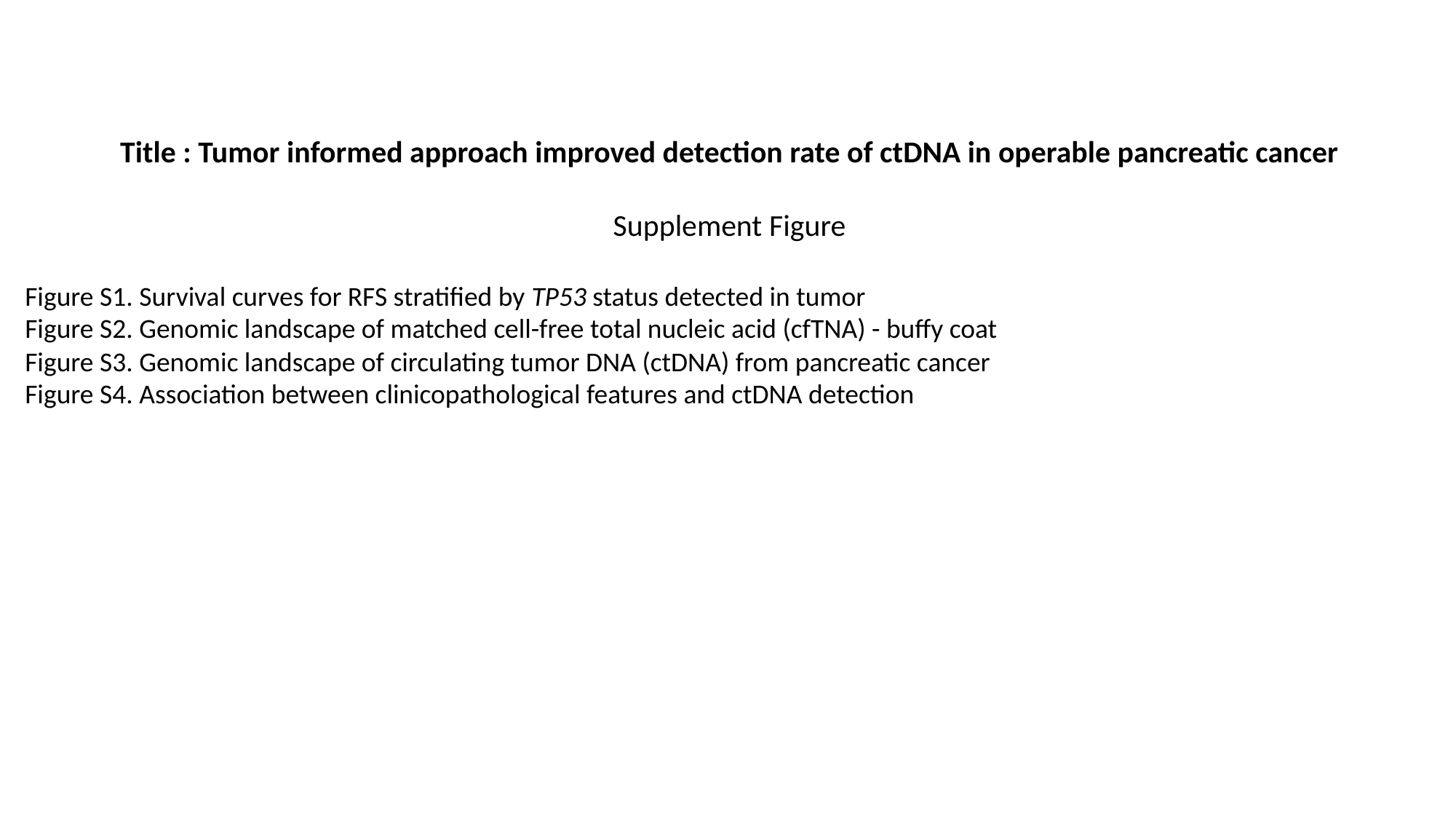

Title : Tumor informed approach improved detection rate of ctDNA in operable pancreatic cancer
Supplement Figure
Figure S1. Survival curves for RFS stratified by TP53 status detected in tumor
Figure S2. Genomic landscape of matched cell-free total nucleic acid (cfTNA) - buffy coat
Figure S3. Genomic landscape of circulating tumor DNA (ctDNA) from pancreatic cancer
Figure S4. Association between clinicopathological features and ctDNA detection

## Slide 2
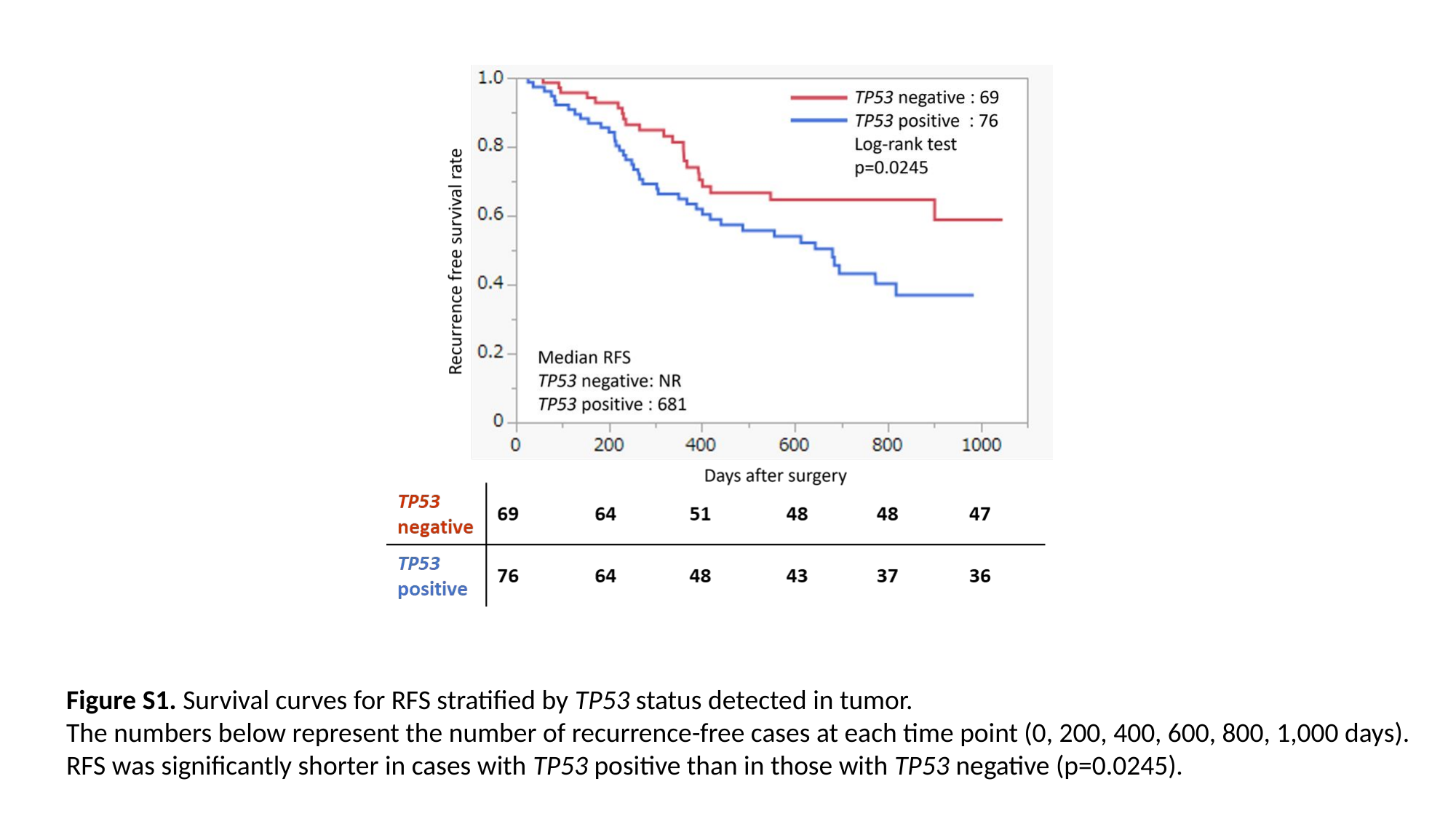

Figure S1. Survival curves for RFS stratified by TP53 status detected in tumor.
The numbers below represent the number of recurrence-free cases at each time point (0, 200, 400, 600, 800, 1,000 days).
RFS was significantly shorter in cases with TP53 positive than in those with TP53 negative (p=0.0245).

## Slide 3
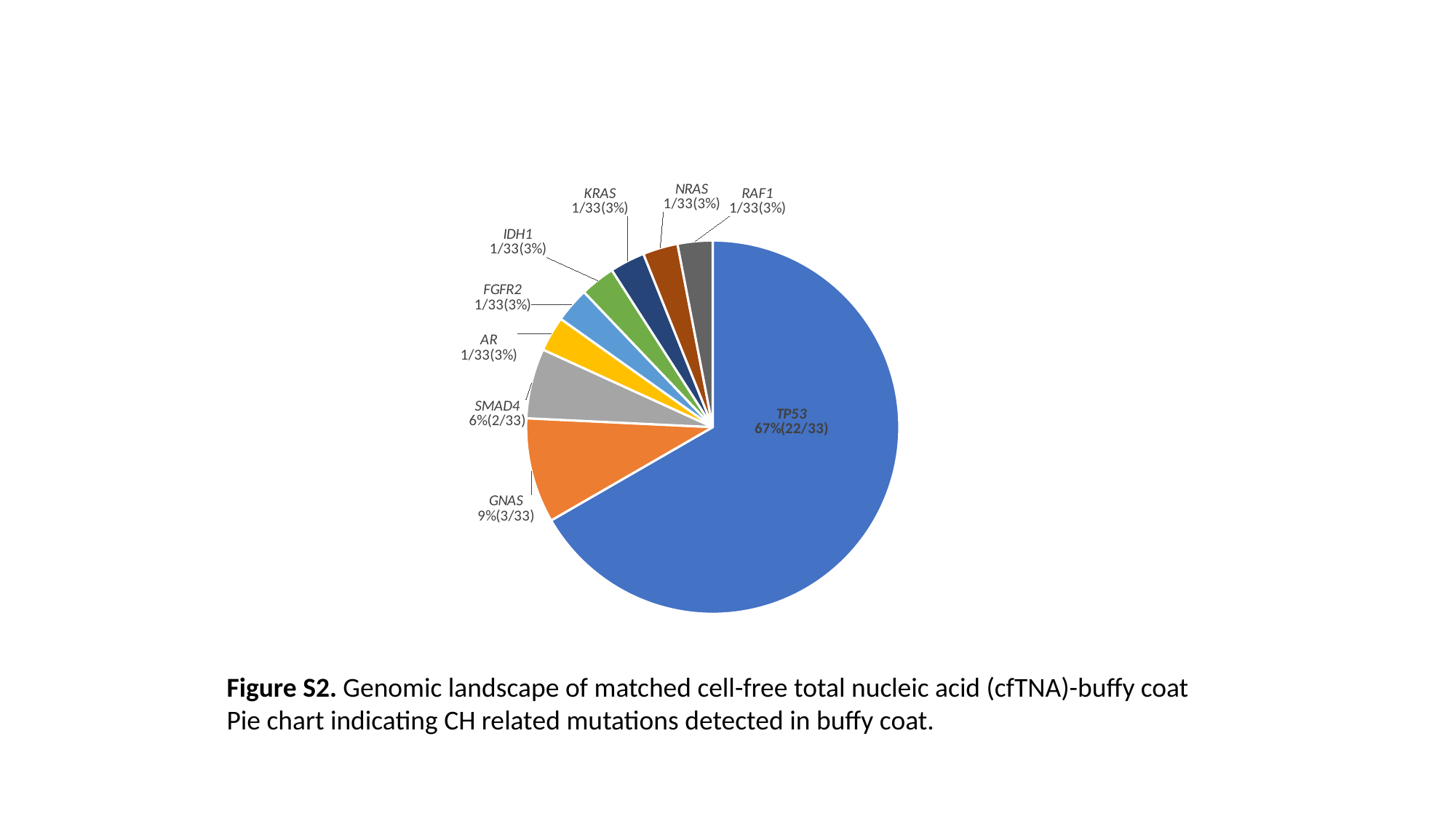

### Chart
| Category | |
|---|---|
| TP53 | 22.0 |
| GNAS | 3.0 |
| SMAD4 | 2.0 |
| AR | 1.0 |
| FGFR2 | 1.0 |
| IDH1 | 1.0 |
| KRAS | 1.0 |
| NRAS | 1.0 |
| RAF1 | 1.0 |Figure S2. Genomic landscape of matched cell-free total nucleic acid (cfTNA)-buffy coat
Pie chart indicating CH related mutations detected in buffy coat.

## Slide 4
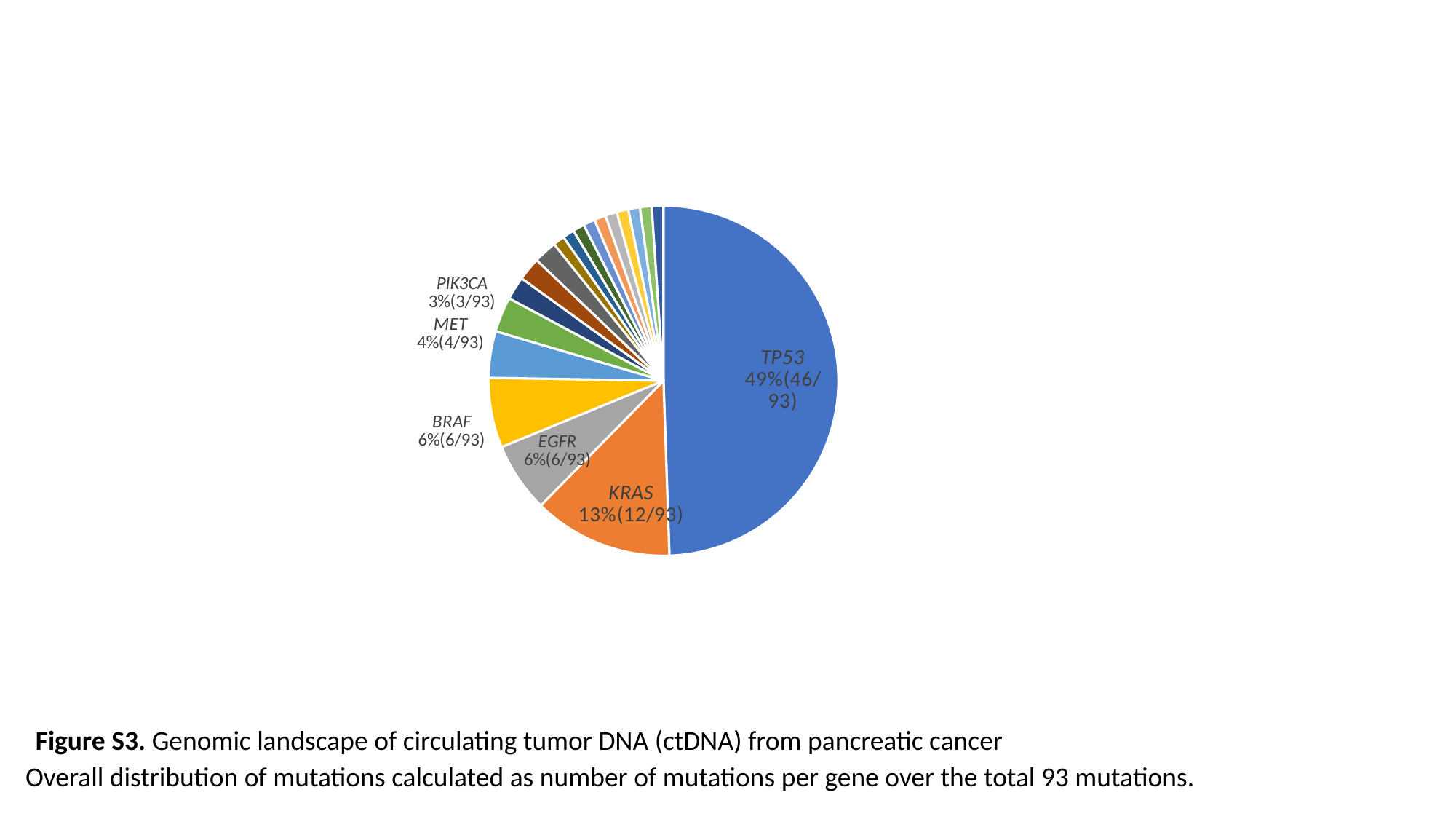

### Chart
| Category | |
|---|---|
| TP53 | 0.4946236559139785 |
| KRAS | 0.12903225806451613 |
| EGFR | 0.06451612903225806 |
| BRAF | 0.06451612903225806 |
| MET | 0.043010752688172046 |
| PIK3CA | 0.03225806451612903 |
| FGFR3 | 0.021505376344086023 |
| GNAS | 0.021505376344086023 |
| SF3B1 | 0.021505376344086023 |
| KIT | 0.010752688172043012 |
| ALK | 0.010752688172043012 |
| ERBB2 | 0.010752688172043012 |
| ESR1 | 0.010752688172043012 |
| MAP2K1 | 0.010752688172043012 |
| MAP2K2 | 0.010752688172043012 |
| NRAS | 0.010752688172043012 |
| NTRK1 | 0.010752688172043012 |
| RET | 0.010752688172043012 |
| SMAD4 | 0.010752688172043012 |Figure S3. Genomic landscape of circulating tumor DNA (ctDNA) from pancreatic cancer
Overall distribution of mutations calculated as number of mutations per gene over the total 93 mutations.

## Slide 5
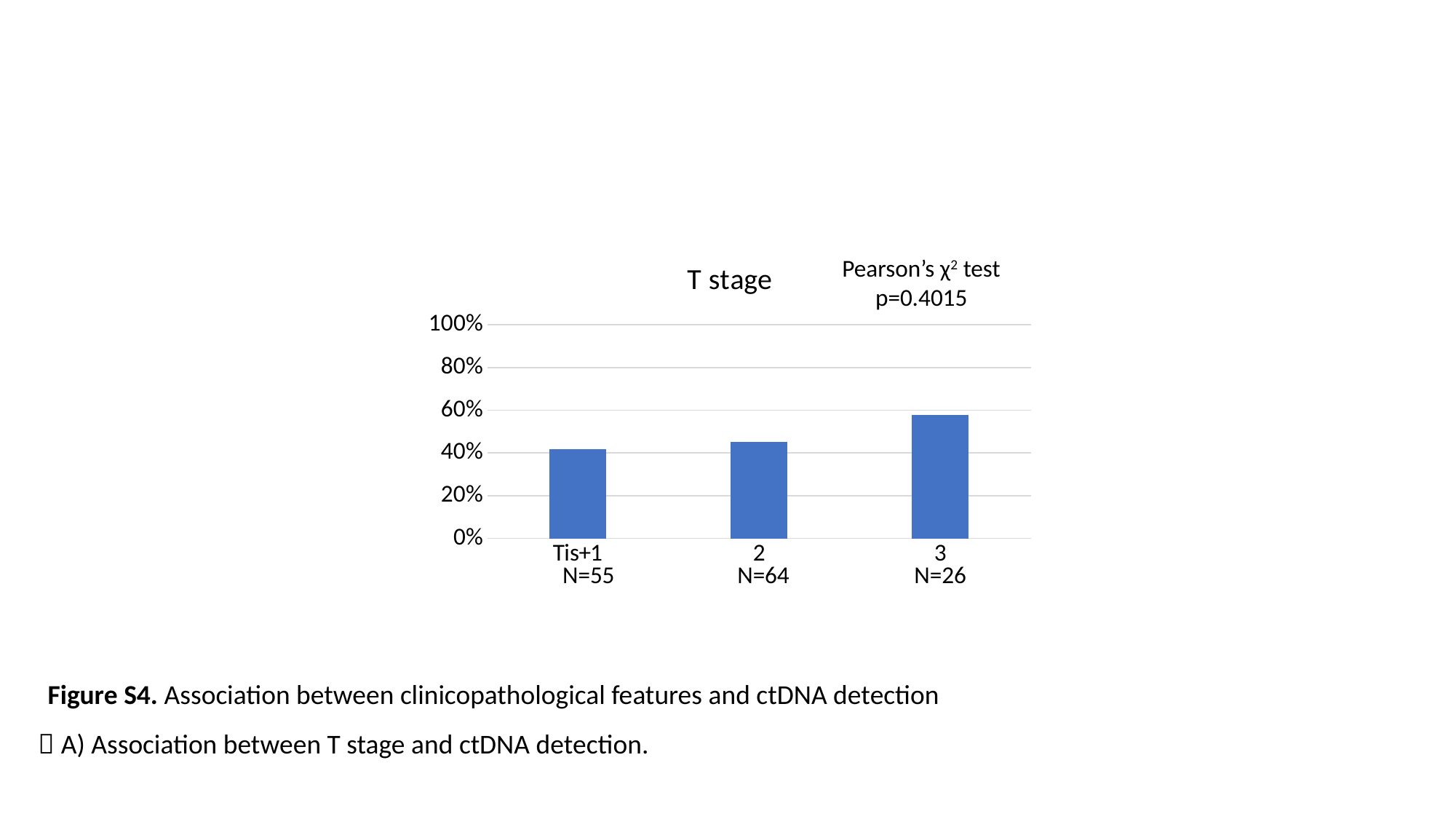

### Chart: T stage
| Category | |
|---|---|
| Tis+1 | 0.41818181818181815 |
| 2 | 0.453125 |
| 3 | 0.5769230769230769 |Pearson’s χ2 test
p=0.4015
N=55
N=64
N=26
Figure S4. Association between clinicopathological features and ctDNA detection
（A) Association between T stage and ctDNA detection.

## Slide 6
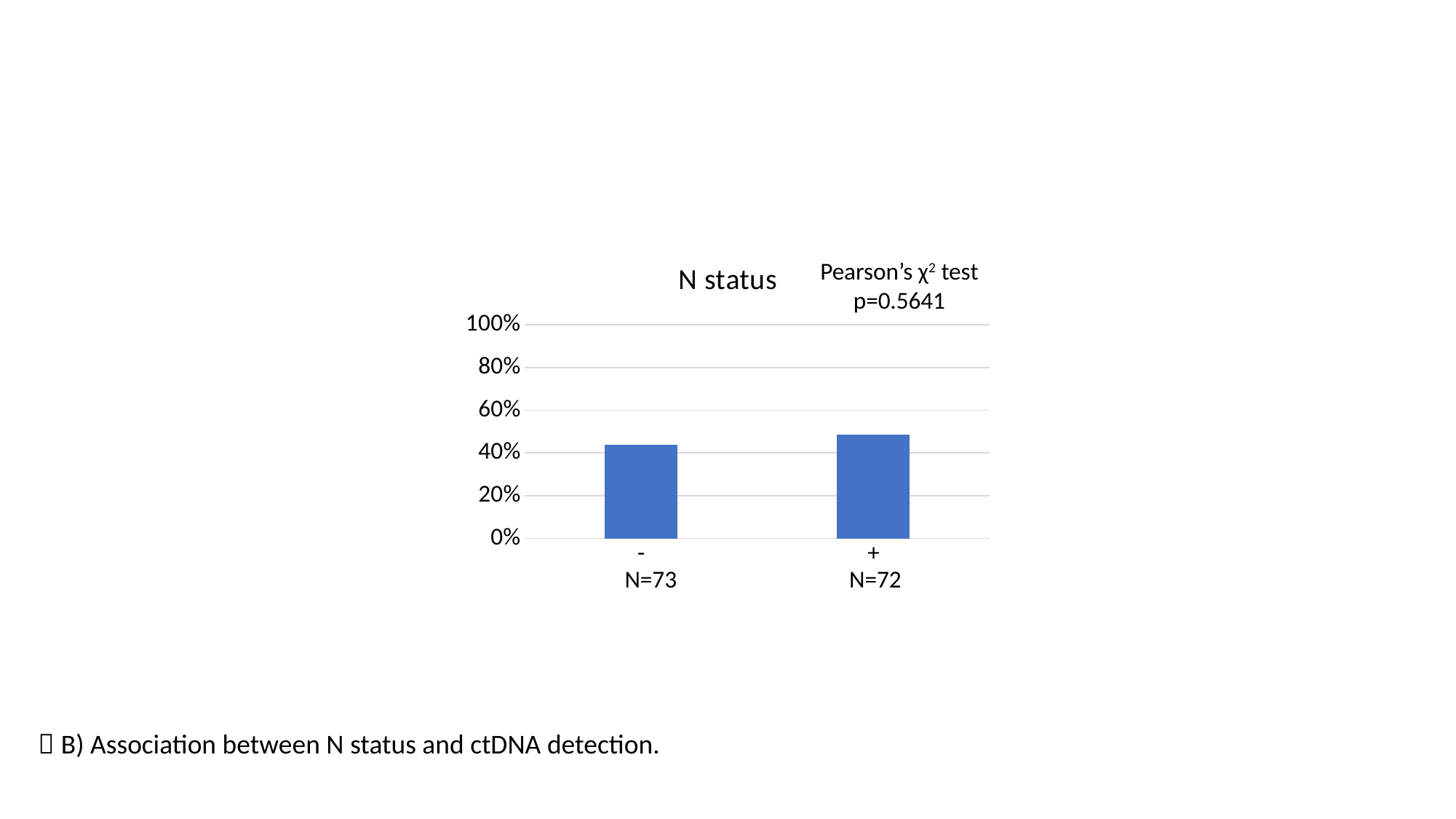

### Chart: N status
| Category | |
|---|---|
| - | 0.4383561643835616 |
| + | 0.4861111111111111 |Pearson’s χ2 test
p=0.5641
N=73
N=72
（B) Association between N status and ctDNA detection.

## Slide 7
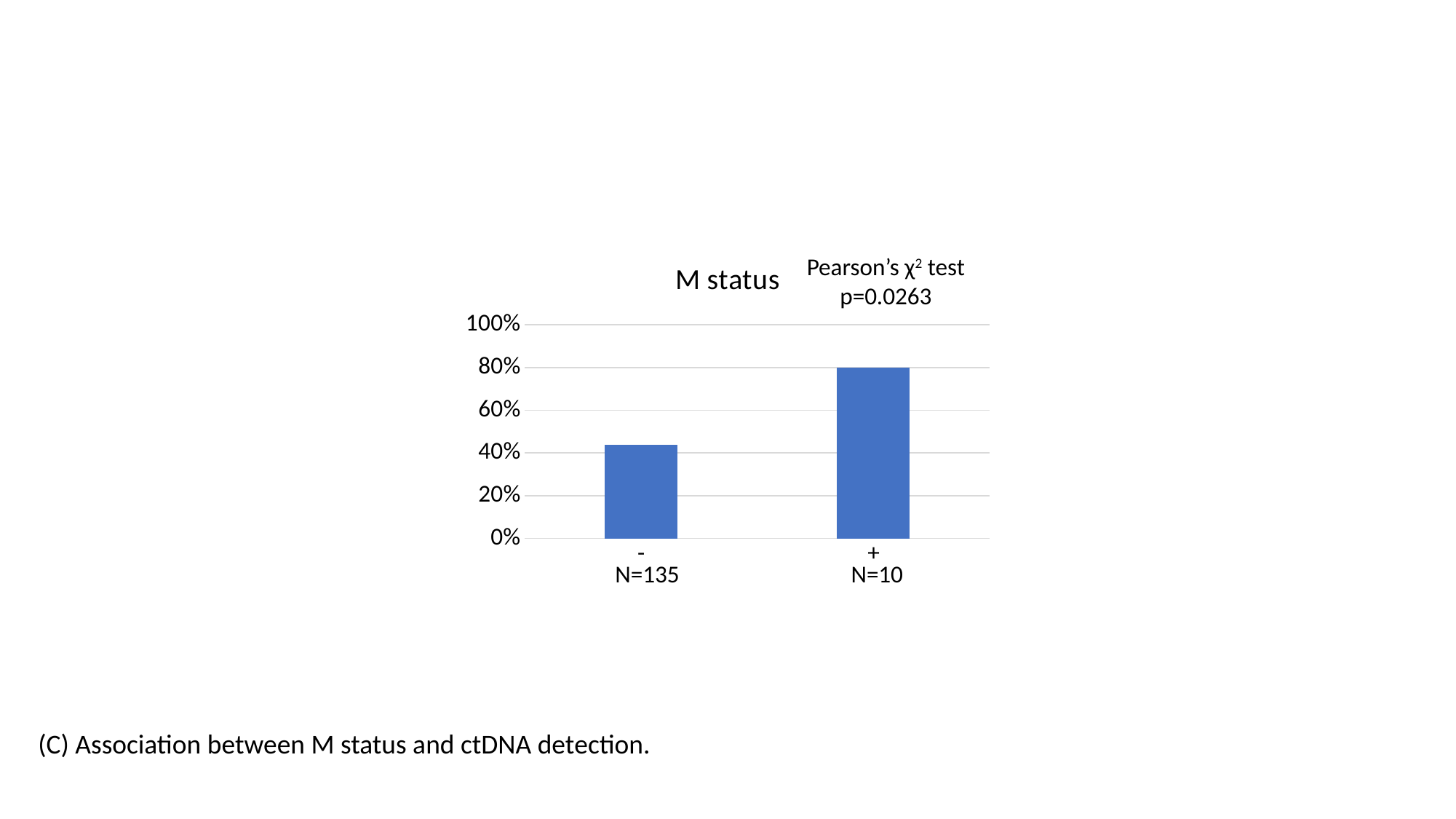

### Chart: M status
| Category | |
|---|---|
| - | 0.43703703703703706 |
| + | 0.8 |Pearson’s χ2 test
p=0.0263
N=135
N=10
(C) Association between M status and ctDNA detection.

## Slide 8
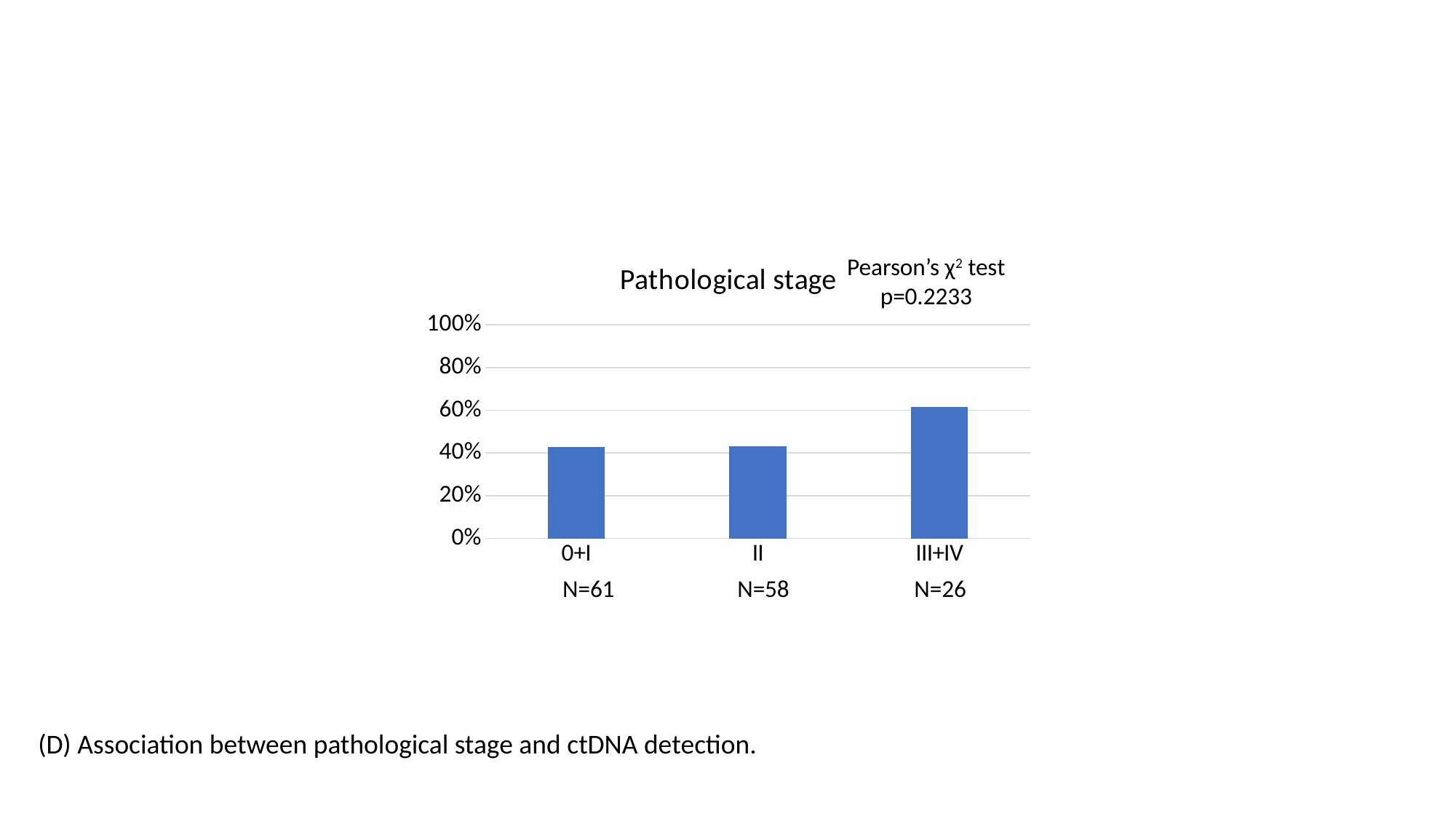

### Chart: Pathological stage
| Category | |
|---|---|
| 0+I | 0.4262295081967213 |
| II | 0.43103448275862066 |
| III+IV | 0.6153846153846154 |Pearson’s χ2 test
p=0.2233
N=61
N=58
N=26
(D) Association between pathological stage and ctDNA detection.

## Slide 9
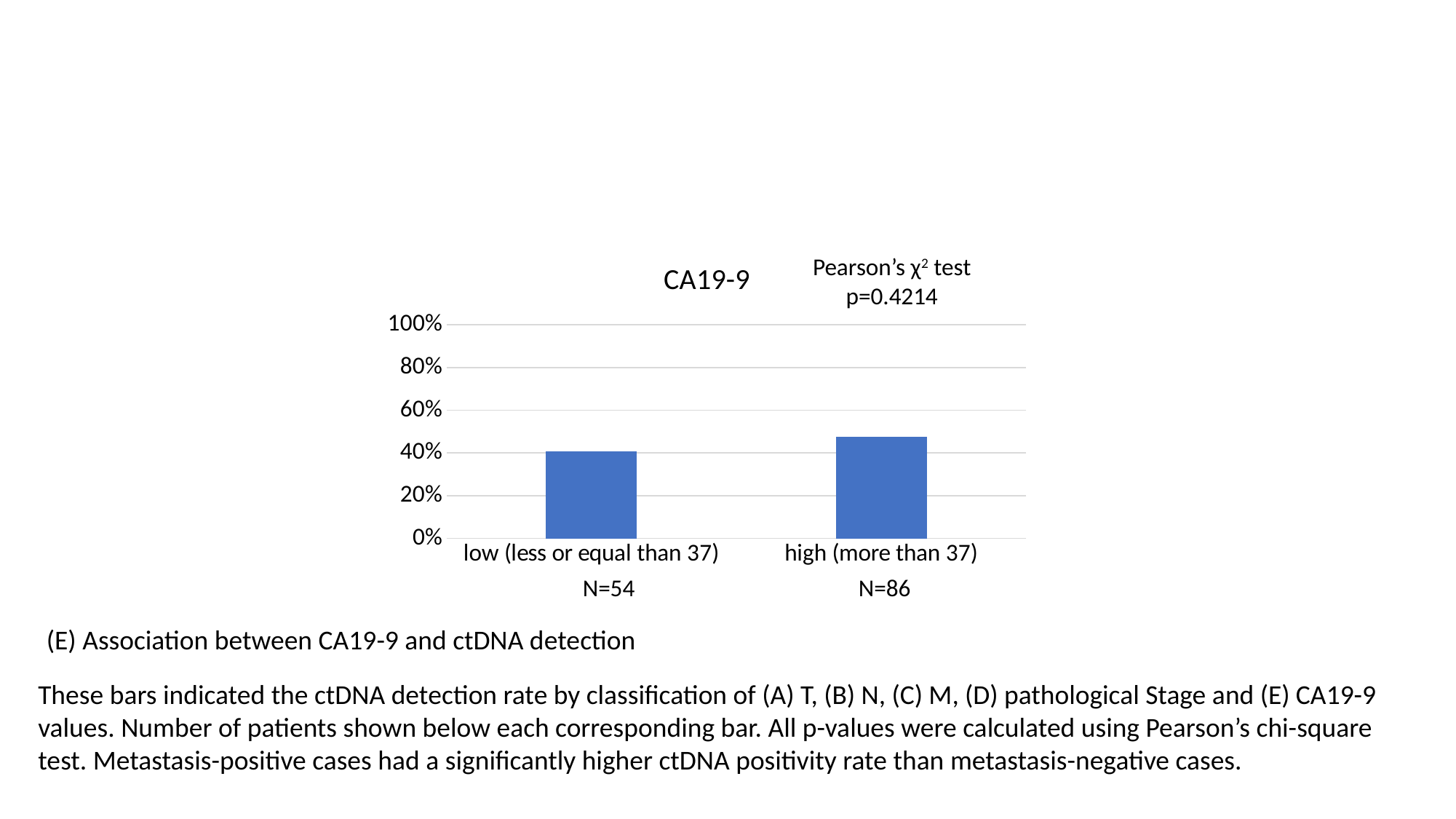

### Chart: CA19-9
| Category | |
|---|---|
| low (less or equal than 37) | 0.4074074074074074 |
| high (more than 37) | 0.47674418604651164 |Pearson’s χ2 test
p=0.4214
N=54
N=86
(E) Association between CA19-9 and ctDNA detection
These bars indicated the ctDNA detection rate by classification of (A) T, (B) N, (C) M, (D) pathological Stage and (E) CA19-9 values. Number of patients shown below each corresponding bar. All p-values were calculated using Pearson’s chi-square test. Metastasis-positive cases had a significantly higher ctDNA positivity rate than metastasis-negative cases.
